# Vibrational Detection of Odorant Functional Groups by *Drosophila melanogaster*

**DOI:** 10.1523/ENEURO.0049-17.2017

**Published:** 2017-10-31

**Authors:** Klio Maniati, Katherine-Joanne Haralambous, Luca Turin, Efthimios M. C. Skoulakis

**Affiliations:** 1 Division of Neuroscience, Biomedical Sciences Research Centre “Alexander Fleming,” Vari 16672, Greece; 2 Department of Chemical Sciences School of Chemical Engineering, National Technical University of Athens, Athens 15780, Greece

**Keywords:** Drosophila, functional groups, olfaction, vibrations

## Abstract

A remarkable feature of olfaction, and perhaps the hardest one to explain by shape-based molecular recognition, is the ability to detect the presence of functional groups in odorants, irrespective of molecular context. We previously showed that *Drosophila* trained to avoid deuterated odorants could respond to a molecule bearing a nitrile group, which shares the vibrational stretch frequency with the CD bond. Here, we reproduce and extend this finding by showing analogous olfactory responses of *Drosophila* to the chemically vastly different functional groups, thiols and boranes, that nevertheless possess a common vibration at 2600 cm^−1^. Furthermore, we show that *Drosophila* do not respond to a cyanohydrin structure that renders nitrile groups invisible to IR spectroscopy. We argue that the response of *Drosophila* to these odorants which parallels their perception in humans, supports the hypothesis that odor character is encoded in odorant molecular vibrations, not in the specific shape-based activation pattern of receptors.

## Significance Statement

To gain mechanistic insights we address predictions of vibrational olfaction in *Drosophila*. We show for the first time that *Drosophila* respond to vibrational frequencies characterizing functional groups. Boranes (-BH) and thiols (-SH) vibrate at the same frequency and flies respond to boranes as if they were thiols strongly suggesting that functional groups of similar enough vibrational frequencies have similar odor character in *Drosophila*. In accord with theoretical predictions, although flies readily detect nitriles, they fail to respond to nitrile groups in molecular configurations that render them undetectable to infrared (IR) spectroscopy. We conclude that the behavioral response of *Drosophila* to these odorant functional groups is consistent with the notion that their character is encoded in their molecular vibrations.

## Introduction

A currently unresolved and enduringly controversial (Block et al., 2015a,b; Turin et al., 2015; Vosshall, 2015) issue in olfaction is the relationship between an odorant’s molecular structure and its odor character. The more commonly accepted theory regards odor as arising from lock-and-key interactions between odorants and the large set of receptors (Buck and Axel, 1991; Zozulya et al., 2001) dedicated to their detection. While plausible, this view has not led to predictions of odor from structure (Sell, 2006), perhaps unsurprisingly given the complexity and the difficulty of predicting structure-activity relations (Rossiter, 1996; Turin and Yoshii, 2003). An alternative theory proposes that odorant receptors detect the molecular vibrational spectrum of odorants (Dyson, 1938; Wright, 1977; DeCou and Meloan, 1995; Havens and Meloan, 1995; Turin, 1996) and the large number of receptors ensures that some will bind to any odorant. Odor character is a property of the molecule itself in this view and is likely reflected in the ability, of humans at least (Klopping, 1971), to detect odorant functional groups in diverse molecular scaffolds. For example, odorants containing -SH smell “sulfuraceous,” while other functional groups give nitrilic, ethereal etc. characters, underlining the tight correlation of group and odor.

We have tested predictions of vibrational theory on flies (Franco et al., 2011; Drimyli et al., 2016) and humans (Gane et al., 2013), using deuterated odorants. Deuteration changes the mass of hydrogens leaving their bonds and molecular shape unchanged, but alters bond vibrational frequencies. Behavioral studies indicate that insects differentiate hydrogenated and deuterated odorants by smell (DeCou and Meloan, 1995; Havens and Meloan, 1995; Franco et al., 2011; Bittner et al., 2012; Gronenberg et al., 2014). Two additional types of experiments confirmed these results and dispelled the notion that discrimination is driven by impurities in the isotopologues. First, recognition of deuteration could be transferred from one H-D odorant pair to another in *Drosophila* (Franco et al., 2011) and the difference in isotopologue-specific receptor activation could go from excitation to inhibition in bees (Paoli et al., 2016). In a more conclusive experiment, *Drosophila* were asked whether nitriles and C-D bonds smell similar and responded by selectively avoiding a novel deuterated odorant after being punished in the presence of a nitrile and vice versa (Franco et al., 2011). These results indicate that flies respond to the odor of the -C≡N and -CD-containing compounds as if equivalent. As -C≡N and -CD have little in common physically and chemically except for a stretch vibration at 2150 cm^−1^, the results strongly support detection of odorant molecular vibrations.

The proposed mechanism for vibrational olfaction is inelastic electron tunneling (Walmsley, 1980; Jaklevic and Lambe, 1966; Adkins and Phillips, 1985; Wang et al., 1993; Turin, 1996). Inelastic electron tunneling spectroscopy (IETS) selection rules govern the intensity with which a given vibration is detected, and are more complex than those of the related Raman and infrared (IR) spectroscopy. Calculation methods for biological IETS mode intensities are still under development (Brookes et al., 2007; Bittner et al., 2012; Solov'yov et al., 2012; Block et al., 2015b), although a first approximation for odor prediction was encouraging (Turin, 2002). Nevertheless, two predictions about IETS spectrometer-perceived vibrational modes should be true by analogy in biological systems: (1) functional groups with similar vibrational frequencies must have similar odors and (2) a functional group vibration invisible in the IR will also be undetected by the olfactory system. Addressing such questions of odorant character obviously requires intact animals and behaviorally salient tasks to drive appropriate responses. Herein we test these two predictions on *Drosophila*. We use the remarkable fact that boranes are the only functional group other than thiols described as having a sulfuraceous character to humans (Stock and Massenez, 1912) and a common stretch vibration in the 2500- to 2600-cm^−1^ range (Anderson and Barker, 1950) to test whether *Drosophila* responds to them as if identical. We test the second prediction using a pair of hydroxynitriles that present either strong or weak -C≡N stretch bands (Socrates, 2001), respectively, and ask whether flies respond to one or both as nitrilic.

## Materials and Methods

### Behavioral assays

*Drosophila* were reared and maintained on standard fly food (Acevedo et al., 2007) at 25°C. Handling before and during the behavioral experiments was as described previously (Franco et al., 2011). *Orco-* anosmic flies carried the null allele *Or83b^1^* of the gene encoding the common subunit of the dimeric OR class of olfactory receptors (Vosshall, 2001), while the *Ir8a*; *Orco-* strain (kindly provided by R. Benton) is also mutant in a Ionotropic Receptor coreceptor (Silbering et al., 2011; Rytz et al., 2013). Anosmic flies were tested similarly to wild-type animals.

For conditioned avoidance behavioral assays, flies of either sex were collected in groups of 50–70 under light CO_2_ anesthesia at least 14 h before experimentation and kept in standard food vials. They were transferred to fresh vials and placed in the dark for acclimatization 1 h before experiments commenced. All experiments used the standard olfactory conditioning T-maze and were performed under dim red light at 25°C as before (Drimyli et al., 2016). Odorants were carried in the maze arms in air streams of 550 ml/min.

Groups of 50–70 animals (=1n) per experimental condition were used in all experiments. Animals (50–70) of the first experimental condition (naïve animals) chose for 90 s between the converging odorants or odorant and air, as specified, without any prior training. At the end of the 90 s, the flies were trapped in the arms (each arm is 10.5 cm in length and 1.5 cm in diameter), counted and performance indexes (PIs) were calculated by subtracting the number of flies in the control (unscented) arm from those in the opposite arm and dividing by the total. Animals of the second experimental condition were placed in the electric grid-lined training arm and were presented with the training odorant (at 550 ml/min) for 1 min while simultaneously receiving 12 electric foot shocks of 90 V DC of 1.2 s each. Subsequently, the flies were transferred to the choice point at the convergence of the two testing arms and allowed to choose for 90 s between the testing odorant and air or two converging odorants as specified. The 50–70 flies of the third experimental condition were trained by footshocks in the presence of the complementary odorant and tested likewise. For example, flies trained either with β-mercaptoethanol or decaborane were tested for their level of avoidance of β-mercaptoethanol versus air. Avoidances of trained animals were compared to those of naïve flies. Moreover, in the complementary experiment, flies trained with β-mercaptoethanol or decaborane were tested for avoidance (or attraction) of decaborane versus air and compared to the performance of naïve animals. Each experimental condition represented in the bar graphs of the figures is constructed by at least seven repetitions each containing 50–70 animals. The total number of animals used to construct each bar is shown in the relevant figure legend.

Because of the potency of β-mercaptoethanol and boranes, we tested whether odors linger on the maze and delivery tubes by testing fly behavior with used maze arms and tubing without odorants. Flies distributed equally in previously used and new arms and tubing indicating that neither odor lingers on the equipment to bias the results.

### Odorants

1-Decanol (Sigma) and d21-decanol (CDN Isotopes) were diluted at 5% in isopropyl myristate (IPM; Sigma) in a total of 1 ml. Citronellyl nitrile (Takasago) and citronellal (Fluka) were diluted at 15% and 10%, respectively, in IPM. H-β-mercaptoethanol (Sigma) and its D_4_-isotopologue (CDN Isotopes) were diluted with water at 1%. β-Mercaptoethanol was diluted at 0.01%, 0.1%, and 25% in water for the response curve of wild-type and anosmic flies. Because decaborane (Carbosynth) is a potent odorant, potentially toxic at higher concentrations, all assays were carried with the use of a protective mask by the experimenter and with frequent pauses between trials to aerate the room and not to overexpose the flies subject to later experiments. Typical experiments used β-mercaptoethanol at 1% in water unless otherwise specified, while decaborane was used as a solid at 50 mg. Benzaldehyde (Sigma) was used at 10% in IPM, and hexanol (Fluka) was used at 10% in IPM. 2-Hydroxybutyronitrile (Sigma) and 3-hydroxybutyronitrile (Sigma) were diluted in IPM at 5%. All odorants were diluted up to 1 ml total volume.

### Statistical analysis

Results were analyzed parametrically using planned comparisons (least squares method) and the statistical program JMP (SAS).

### Density-functional calculations

Conformations and vibrational spectra of molecules were calculated with Amsterdam Density Functional (www.scm.com) and Spartan 14 (www.wavefun.com) at either PBE-DZP or B3LYP-6-311 level of theory.

## Results

*Drosophila* trained to selectively avoid a deuterated compound, but not its hydrogen counterpart, also avoid preferentially a nitrile group, whose vibration around 2150 cm^−1^ is shared with the C-D stretch (Franco et al., 2011). Because of the significance of this result, which is highly unlikely due to contaminants or differences in the physical properties of isotopologues, we confirmed it independently using a different combination of odorants and more importantly, the independent, simpler training paradigm described recently (Drimyli et al., 2016). In this conditioned aversion paradigm, flies shocked in the presence of an odorant are asked whether they recognize features of the training compound in a different odorant used for testing.

**Figure 1.**
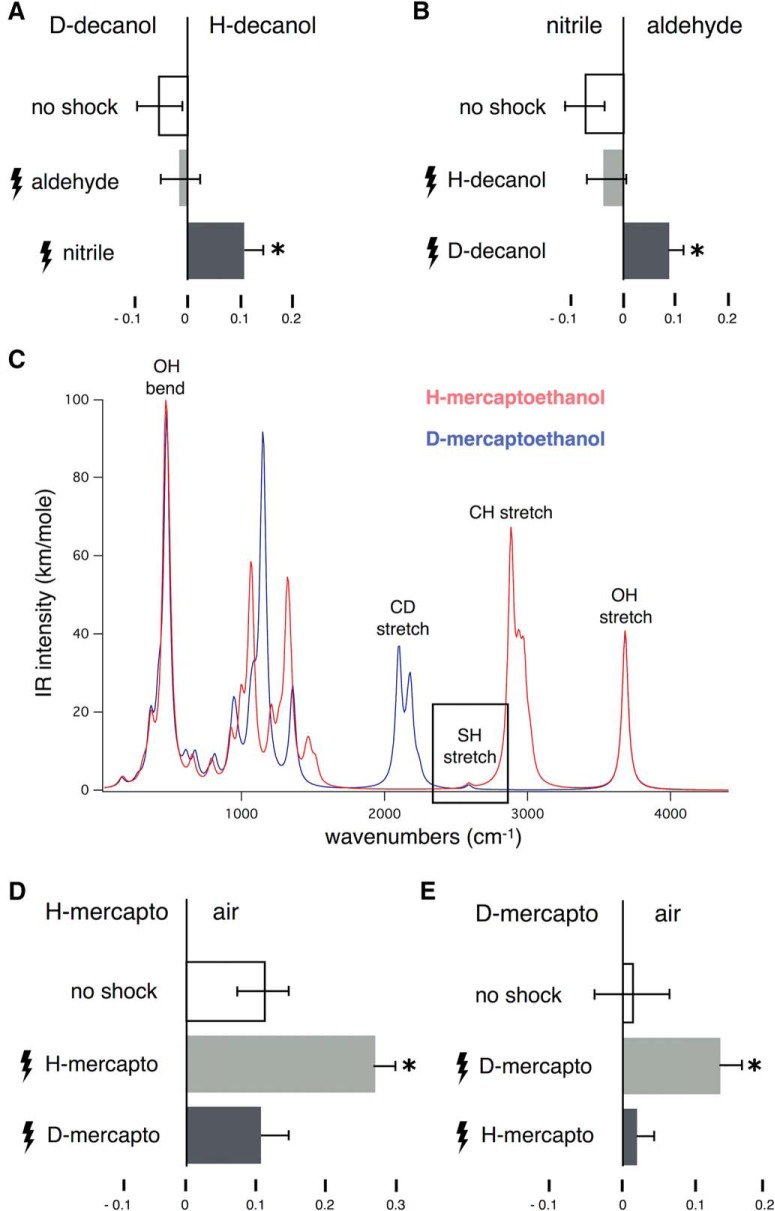
Odorant vibrations guide conditioned behavior in *Drosophila*. All graphs represent the mean relative distribution of flies in the arms of the maze ± SEM after the indicated treatment. The odorants or air converging in the center of the maze during the test phase, are indicated above each graph. “No shock” condition refers to groups of naïve flies responding to the indicated odor(s). Other groups of flies were trained by repeated electric footshocks (lightning bolts) in the presence of the indicated odorants per condition. All significance values are calculated relative to the performance of naïve animals (open bars), unless noted otherwise, by LSM contrast analysis and are indicated on the graphs with a single star. ***A***, Transfer of olfactory learning between citronellyl nitrile (nitrile) and D-decanol. Initial ANOVA indicated significant differences (*F*_(2,40)_ = 5.5868, *p* < 0.007). Flies trained to avoid citronellal (aldehyde) did not significantly avoid (*p* = 0.4267) either test odorant (light gray bar), whereas flies shocked against nitrile (dark gray bar) avoided D-decanol significantly (*p* = 0.003). *n* > 10 for all conditions, with a total of 700–900 flies per condition. ***B***, In the converse experiment, ANOVA indicated significant differences (*F*_(2,38)_ = 4.8303, *p* < 0.01). Subsequent contrast analysis revealed that flies trained to avoid H-decanol did not change their spontaneous behavior (light gray bar, *p* = 0.5029). In contrast, animals trained to avoid D-decanol presented significant avoidance to the nitrile (dark gray bar, *p* = 0.0073). *n* > 10 for all conditions, with a total of 700–1000 flies per condition. ***C***, Computed I.R spectra of H- and D4-β-mercaptoethanol indicating the frequencies of the relevant C–D, -SH, and C–H stretches. ***D***, Conditioned H- and D4-β-mercaptoethanol discrimination. Initial ANOVA indicated significant differences (*F*_(2,43)_ = 6.2144, *p* < 0.004). Flies trained to selectively avoid the H isotopologue augmented their avoidance of H-β-mercaptoethanol over that of naïve animals (*p* = 0.0041), but not for D4-β-mercaptoethanol (*p* = 0.9536). The performances of the two groups trained to different isotopologues were also significantly different (*p* = 0.0040). *n* ≥ 14 for all conditions, with a total of 900–1000 flies per condition. ***E***, In the converse experiment, animals trained with each of the isotopologues were tested against D-β-mercaptoethanol. ANOVA indicated significant differences (*F*_(2,31)_ = 3.6338, *p* < 0.03). Animals trained with the D isotopologue enhanced their avoidance of D-β-mercaptoethanol (*p* = 0.0259), but not of H-β-mercaptoethanol (*p* = 0.9236). The performance of the two groups is different (*p* = 0.0255). *n* > 10 for all conditions, with a total of 600–700 flies per condition.

At the concentration used, naïve flies presented a very mild attraction to D-decanol, or aversion from its normal isotopologue converging in the center of the testing maze (Fig. 1*A*, open bar). A similar non-biased response was observed when flies were conditioned with citronellal, which does not possess any similar molecular features to either decanol isotopologue (Fig. 1*A*, light gray bar). This confirms that the testing isotopologues do not differ in volatility, contaminants or other properties that might bias the results. However, animals conditioned to preferentially avoid citronellyl nitrile, selectively avoided the deuterated decanol isotopologue (Fig. 1*A*). Conversely, animals shocked in the presence of D-decanol, avoided citronellyl nitrile selectively over the aldehyde (Fig. 1*B*). Again, the naïve responses to the test odorants and those after conditioning with the aldehyde were statistically identical, in agreement with prior reports (Franco et al., 2011). The results are consistent with *Drosophila* recognizing the 2150 cm^−1^ vibration of the C-D or the C≡N stretch and use it to guide behavioral choices in two independent paradigms (Franco et al., 2011; herein).

In previous experiments, flies discriminated efficiently between otherwise identical odorants (isotopologues) except for the presence of the C-D and C-H vibrations at 2150 and 2900 cm^−1^, respectively. Can this discrimination occur in the presence of another dominant odor character? To address this question, we chose the S-H vibration, which at 2550 cm^−1^ lies between those of the -CH and -CD/-C≡N (Fig. 1*C*) and possesses a distinct sulfur odor character. Thus, we asked whether flies could discriminate the simple thiol, β-mercaptoethanol from its d4-isotopologue (D-mercapto).

To avoid potential differences in volatility biasing the results, we used the footshock-mediated augmentation of avoidance assay (Drimyli et al., 2016). In this assay, naïve avoidance or attraction to an odorant is modified by concurrent presentation of footshocks, but a second odorant (or isotopologue) not associated with footshocks is not used as control. Therefore, the assay is not associative in the strict sense and does not require the isotopologues to be tested against each other at the choice point such that their volatility or other properties may skew experimental outcomes. If a deuterated compound is used for training, flies augment their avoidance of the same odorant used for testing compared to their naïve response toward it. On the other hand, if flies differentiate between isotopologues, they would not augment their avoidance of the deuterated odorant in testing, if shocked in the presence of its normal counterpart during training (Drimyli et al., 2016). If the flies cannot differentiate two odorants or isotopologues, then they are expected to respond equally to the either odorant on testing respective of which was coupled to footshocks during training (trained against).

Normal β-mercaptoethanol is mildly aversive, while its d4-isotopologue is neutral to *Drosophila* at 1% dilution (Fig. 1*D*,*E*, no shock). Aversion was strongly augmented when β-mercaptoethanol, but not the deuterated isotopologue, was coupled to footshocks (Fig. 1*D*). Conversely, flies trained to avoid the deuterated isotopologue augmented their avoidance specifically to d4-β-mercaptoethanol, but did not change their behavior toward the normal odorant (Fig. 1*E*). Therefore, the -SH stretch vibration does not interfere with -CH versus -CD discrimination. Interestingly, animals trained to avoid the d4-β-mercaptoethanol responded to the normal isotopologue as if they were naïve (Fig. 1*D*), and similarly, animals shocked in the presence of normal β-mercaptoethanol did not present enhanced avoidance of the deuterated isotopologue (Fig. 1*E*). This is surprising given that the -SH character is dominant and common to both isotopologues, at least to the human nose. However, the results are consistent with the notion that flies differentiate the isotopologues solely on the basis of their distinct -CH and -CD vibrations and appear to ignore their apparently common -SH odor character.

**Figure 2.**
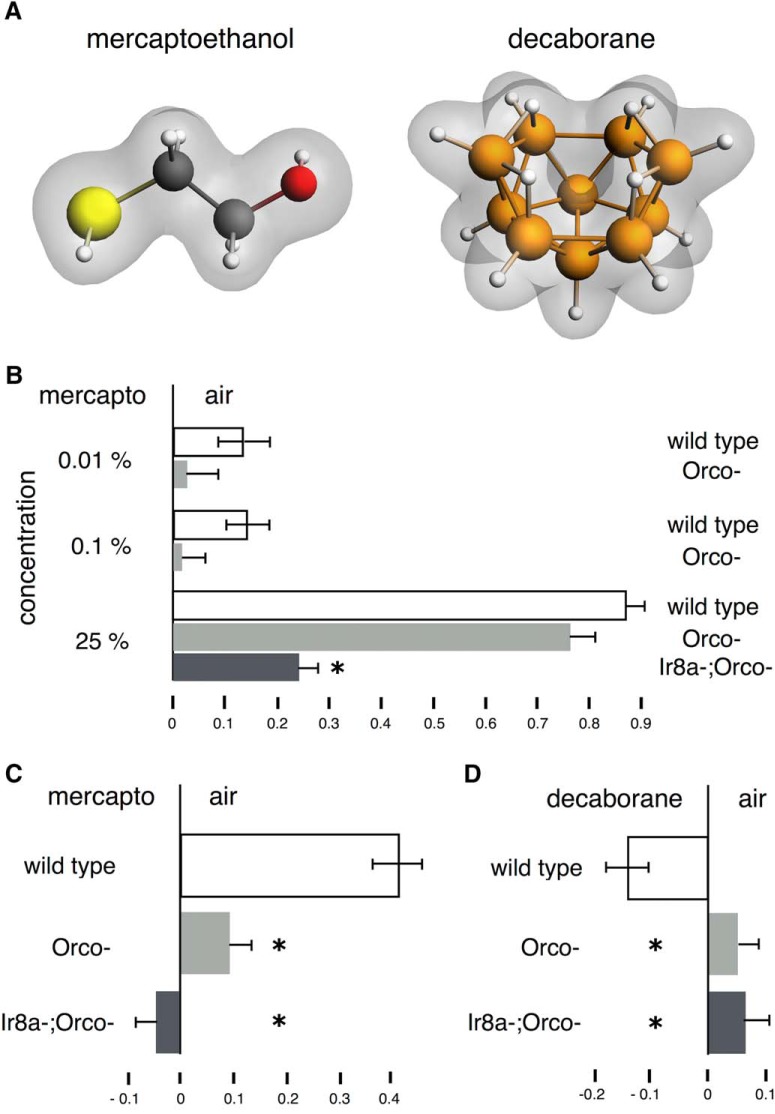
Responses to β-mercaptoethanol and decaborane in wild-type *Orco^−^* and *Ir8a^−^* *Orco^−^* double mutants. ***A***, The high molecular divergence of β-mercaptoethanol and decaborane. ***B–D***, All graphs represent the mean relative distribution of flies in the arms of the maze ± SEM after the indicated treatment. Groups of flies were exposed to the indicated odorants per condition. Significance values are calculated by LSM contrast analysis and are indicated on the graphs with a single star. ***B***, Responses of wild-type and anosmic flies to a range of β-mercaptoethanol concentrations. Wild-type flies are shown in white bars, *Orco^−^* flies are shown in light gray bars and *Ir8a;Orco*- flies are shown in dark gray bars. ANOVA indicated significant differences (*F*_(6,87)_ = 39.7679, *p* < 0.0001). At the 0.01% concentration, the difference between wild-type and *Orco^−^* flies is not significant (*p* = 0.2394), but at the 0.1% concentration, the difference is significant (*p* = 0.0454). At the 25% concentration the response of wild-type and *Orco^−^* flies is not significantly different (*p* = 0.1938) but that of *Ir8a^−^;Orco^−^*is (*p* < 0.0001). *n* ≥ 8 for all conditions, with a total of 600-1000 flies per condition. ***C***, ANOVA indicated significant differences (*F*_(2,35)_ = 38.3487, *p* < 0.0001), which were revealed due to the significant difference in avoidance of β-mercaptoethanol by wild-type and both *Orco^−^* and *Ir8a;Orco-* mutants (both *p* < 0.0001). The difference between *Orco^−^* and *Ir8a;Orco-* mutants is also significant (*p* = 0.0445). *n* ≥ 10 for all conditions, with a total of 700-900 flies per condition. ***D***, ANOVA indicated significant differences (*F*_(2,62)_ = 2.5955, *p* < 0.0830), underlying the significant difference in attraction to decaborane between wild-type, *Orco^−^* mutants (*p* = 0.0518), and *Ir8a;Orco- (p* = 0.0341). The difference between *Orco^−^* and *Ir8a;Orco-* mutants is not significant (*p* = 0.8832). *n* ≥ 12 for all conditions, with a total of 800–1400 flies per condition.

Although, as suggested previously (Franco et al., 2011; Drimyli et al., 2016) and by the abovementioned results, deuteration precipitates a salient change in odor character, the contribution of the vibrations characterizing the different functional groups has not been explored. However, IETS rules predict that functional groups with the same or very similar vibrational frequency should have similar odor characters. To test this formally, we initially capitalized on the impressive commonality in odor character to the human nose at least, between thiols and boranes. Although structurally dissimilar (Fig. 2*A*) and despite their lack of -SH groups, boranes nevertheless have been reported to smell sulfuraceous from the time of their discovery and characterization (Stock and Massenez, 1912). We hypothesized that this similarity in odor character may be consequent of the only property boranes and thiols share, namely the stretch vibration in the 2500- to 2600-cm^−1^ range. Therefore, we aimed to test this notion on the commonality of odor character between -SH and -BH on *Drosophila*.

Given the abovementioned results (Fig. 1*D*,*E*), we wondered whether flies smell and respond to -SH. We used β-mercaptoethanol, which at 0.01–0.1% (Fig. 2*B*) and 1% (Fig. 2*C*), is mildly to moderately aversive to wild-type *Drosophila*, an effect that requires the OR class of receptors, because mutations in their common subunit ORCO eliminate the response (Fig. 2*B*,*C*). However, high concentrations (25%) of β-mercaptoethanol elicited elevated avoidance both in wild-type and Orco^−^ flies, an effect that was attenuated if the IR8a ionotropic receptor coreceptor was lost (*Ir8a^−^*, *Orco^−^*; Fig. 2*B*). These results indicate that at the 0.01–1% range (Fig. 2*B*,*C*), β-mercaptoethanol addresses primarily the OR class of receptors, whereas at high concentrations (i.e., 25%), IR-type receptors are also engaged and contribute to the strong avoidance behavior (Fig. 2*B*). Decaborane was mildly attractive to *Drosophila* and again this effect was eliminated in *Orco^−^* and *Ir8a^−^*, *Orco^−^* animals (Fig. 2*D*), indicating that it also addresses the OR class of receptors.

**Figure 3.**
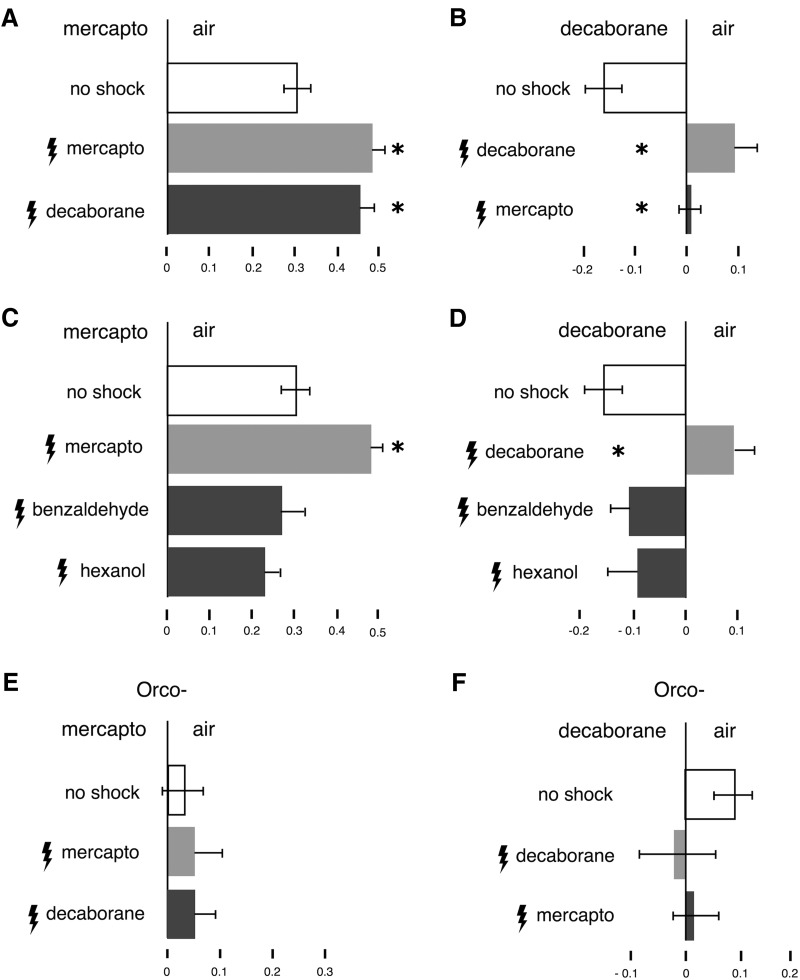
After conditioning *Drosophila* do not discriminate decaborane from β-mercaptoethanol. All graphs represent the mean relative distribution of flies in the arms of the maze ± SEM after the indicated treatment. Significant differences are calculated relative to the performance of naïve animals (open bars) by LSM contrast analysis and are indicated on the graphs with a single star. ***A***, ANOVA indicated significant differences (*F*_(2,59)_ = 8.5292, *p* = 0.0006). Flies shocked in the presence of β-mercaptoethanol augmented their avoidance toward it (light gray bar, *p* = 0.0005) and surprisingly a similar augmentation was shown by animals shocked in the presence of decaborane (dark bar, *p* = 0.0006). Indeed, the performances of the two differently trained groups were not significantly different (*p* = 0.5915). *n* ≥ 12 for all conditions, with a total of 900-2000 flies per condition. ***B***, In the converse experiment ANOVA indicated significant differences (*F*_(2,30)_ = 15.3343, *p* < 0.0001). Flies shocked in the presence of decaborane avoid it instead of being attracted to it as naïve animals do (light gray bar, *p* < 0.0001) and a similar response is observed in β-mercaptoethanol-trained animals (dark bar, *p* = 0.0006). The two trained groups do not show significantly different responses (*p* = 0.1150). *n* ≥ 8 for all conditions, with a total of 500–900 flies per condition. ***C***, Animals shocked against benzaldehyde (10%) or (10%) hexanol do not change their spontaneous response to β-mercaptoethanol. ANOVA indicated significant differences (*F*_(3,60)_ = 7.9061, *p* = 0.0002). Subsequent contrast analysis revealed significant differences in the performance of β-mercaptoethanol-trained animals (light gray bars) versus those trained with benzaldehyde (*p* = 0.0006) and hexanol (*p* < 0.0001). The responses of flies trained with benzaldehyde and hexanol (dark bars) were not significantly different (*p* = 0.3997 and *p* = 0.0813, respectively) from naïve animals (open bars). *n* ≥ 9 for all conditions, with a total of 700–2000 flies per condition. ***D***, Animals shocked against benzaldehyde or hexanol do not change their spontaneous response to decaborane. ANOVA indicated significant differences (*F*_(3,43)_ = 4.9331, *p* = 0.0052). Contrast analysis revealed significant differences in the performance of decaborane-trained animals (light gray bars) versus those trained with benzaldehyde (*p* = 0.0003) and hexanol (*p* = 0.0004). The responses of flies trained with benzaldehyde and hexanol (dark bars) were not significantly different (*p* = 0.2403 and *p* = 0.1561, respectively) from naïve animals (open bars). *n* ≥ 8 for all conditions, with a total of 500–900 flies per condition. ***E***, ANOVA did not indicate significant differences (*F*_(2,26)_ = 0.1105, *p* = 0.8959). Anosmic animals shocked in the presence of β-mercaptoethanol or in the presence of decaborane did not augment their response to either (light gray bar, *p* = 0.6792 and dark gray bar, *p* = 0.7059, respectively). Furthermore, the performances of the two differently trained groups of flies were not significantly different (*p* = 0.9624). *n* ≥ 8 for all conditions, with a total of 600-700 flies per condition. ***F***, In the converse experiment, ANOVA also did not indicate significant differences (*F*_(2,48)_ = 0.7551, *p* = 0.4757). Flies shocked in the presence of decaborane or β-mercaptoethanol did not change their irresponsiveness toward decaborane (light gray bar, *p* =0.24 and dark bar, *p* = 0.42, respectively). The responses of the two differently trained groups were not significantly different (*p* = 0.69). *n* ≥ 8 for all conditions, with a total of 800–1000 flies per condition.

Flies trained by footshocks to avoid β-mercaptoethanol, augmented significantly their naive avoidance (Fig. 3*A*, light gray bar). Significantly, animals shocked in the presence of decaborane avoided β-mercaptoethanol as if they were trained to avoid the latter (Fig. 3*A*, dark gray bar). In the complementary experiment, pairing decaborane to electric shock resulted in its avoidance instead of the attraction presented by naive animals (Fig. 3*B*, light gray bar). In accord, flies trained to avoid β-mercaptoethanol also did not show attraction to decaborane (Fig. 3*B*, dark gray bar). Because the -BH and -SH functional groups possess distinct sizes and shapes (Fig. 2*A*), it is unlikely that these properties can account for the similar conditioned responses to the thiol and borane. Therefore, because they lack any other common features, we conclude that flies likely use the common thiol/borane 2500–2600 cm^−1^ vibration to guide their conditioned avoidance behavior.

Alternatively, once shocked in the presence of any odor, flies could simply avoid any subsequent odor presented to them (generalize) and escape toward the unscented maze arm. To address this possibility, we used the unrelated aversive odorant benzaldehyde (naïve benzaldehyde avoidance PI = 0.615998 ± 0.090367, *n* = 8) and the neutral odorant hexanol (naïve hexanol avoidance PI = −0.0070045 ± 0.05384269, *n* = 7), for training and tested subsequent avoidance toward the thiol and borane. Flies shocked in the presence of β-mercaptoethanol showed augmentation of their naive thiol avoidance (Fig. 3*C*, light gray bar). However, flies trained to avoid benzaldehyde or hexanol did not enhance their avoidance, but rather behaved as if naïve toward β-mercaptoethanol (Fig. 3*C*, dark gray bars) and similarly naïve toward decaborane (Fig. 3*D*). Although benzaldehyde is one of the most effective natural repellents (Knaden et al., 2012) and the flies are shocked in its presence, they nevertheless do not generalize by augmenting their avoidance toward any odorant presented to them. This argues strongly for the specificity of the footshock-mediated avoidance augmentation assay and validates the behavioral responses of the flies. Avoidance augmentation is observed only when a common element is present in the training and testing odor. This strongly supports the notion that *Drosophila* deem the odors equivalent and respond similarly to them. Significantly, since β-mecrcaptoethanol and decaborane do not share any other physical or chemical characteristics, these results are consistent with the notion that this behavioral equivalence is a consequence of the -SH and -BH common vibration frequency at 2500-2600 cm^−1^. In contrast, *Orco^−^* mutants, which do not avoid β-mercaptoethanol (Fig. 3*E*), or are attracted to decaborane (Fig. 3*F*), fail to augment their minimal responses to these odorants when paired with footshocks (Fig. 3*E*,*F*). Consequently, *Orco^−^* flies fail the avoidance augmentation assay unlike wild-type animals (Fig. 3*E*,*F* vs *A*,*B*). This confirms the notion that wild-type flies use OR-type receptors to respond to β-mercaptoethanol and decaborane.

**Figure 4.**
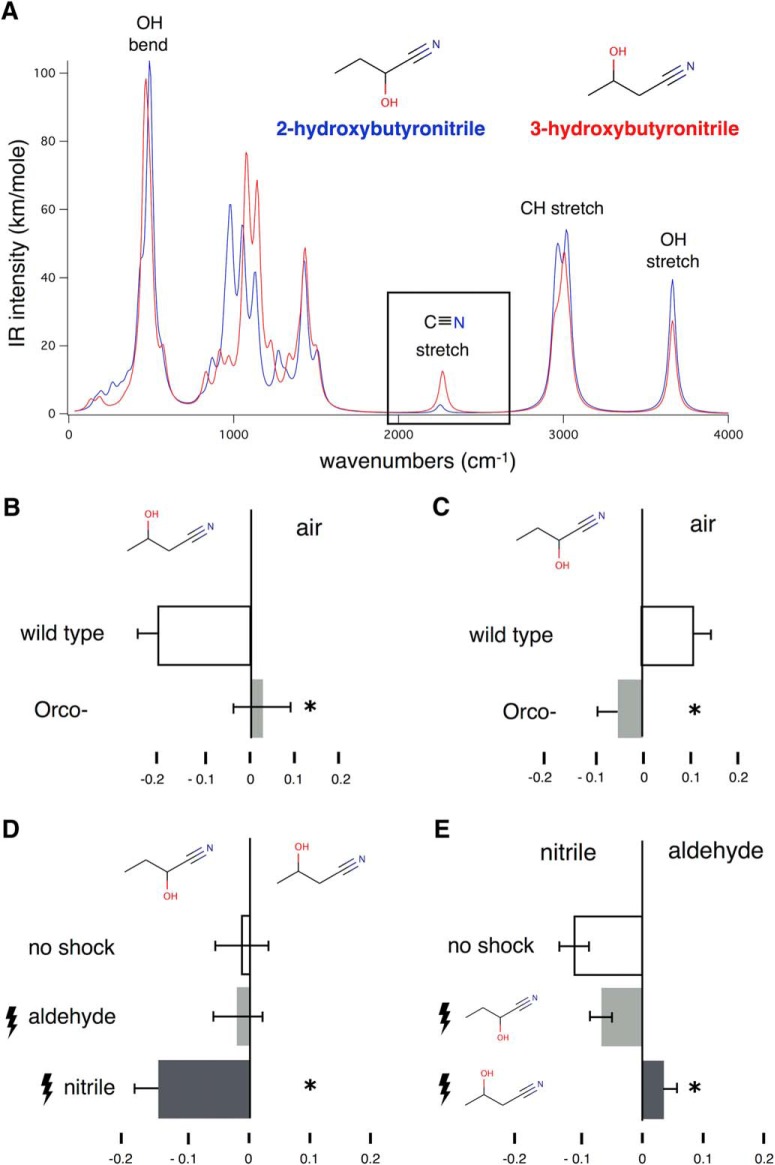
Differential responses to nitrile in the context of different hydroxynitriles. ***A***, Computed I.R spectra of 2- and 3-hydroxybutyronitrile. The location of the -OH group that changes the intensity of the -C≡N stretch (boxed) is shown. Graphs represent the mean relative distribution of flies in the arms of the maze ± SEM after the indicated treatment per condition. Groups of flies were trained by repeated electric footshocks (lightning bolts) in the presence of the indicated odorants per condition. All significance values are calculated relative to the performance of naïve animals (open bars) by LSM contrast analysis and are indicated on the graphs with a single star. ***B***, ANOVA indicated significant differences (*F*_(1,19)_ = 7.6564, *p* < 0.012), underlying the significant difference (*p* = 0.0076) in attraction to 3-hydroxybutyronitrile by wild-type and *Orco^−^* mutants. *n* ≥ 7 for all conditions, with a total of 400-500 flies per condition. ***C***, ANOVA indicated significant differences (*F*_(3,39)_ = 6014, *p* < 0.002), underlying the significant difference (*p* = 0.0136) in avoidance of 2-hydroxybutyronitrile by wild-type and *Orco^−^* mutants. *n* ≥ 11 for all conditions, with a total of 700-800 flies per condition. ***D***, ANOVA indicated significant differences (*F*_(2,39)_ = 3.4240, *p* < 0.04), when flies were differentially trained with citronellal (aldehyde). Flies trained with the aldehyde did not respond differentially (light gray bar, *p* = 0.9052) when tested against the two hydroxynitriles. In contrast, animals trained with citronellyl nitrile (nitrile) avoided preferentially 3-hydroxybutyronitrile (dark bar, *p* = 0.0208). The performance of the differently trained groups was significantly different (*p* = 0.0439). *n* ≥ 11 for all conditions, with a total of 600-900 flies per condition. ***E***, In the complementary experiment ANOVA indicated significant differences (*F*_(2,24)_ = 11.3598, *p* < 0.0004). Flies trained with 2-hydroxybutyronitrile did not exhibit responses different from naïve flies (light gray bar, p= 0.1993). However, flies shocked in the presence of 3-hydroxybutyronitrile avoided the nitrile significantly (dark gray bar, *p* = 0.0001). The performance of the two differently trained groups was significantly different (*p* = 0.0058). *n* ≥ 11 for all conditions, with a total of 500-600 flies per condition.

These results also suggest that if the characteristic vibration of a functional group is diminished, or eliminated then it will no longer be recognized as of the same or sufficiently similar odor character with another molecule bearing the same functional group. To address this hypothesis, we focused on cyanohydrins, compounds in which a -C≡N group is attached to the same carbon as an -OH group (Fig. 4*A*). To humans most cyanohydrins lack the metallic odor character of -C≡N-containing compounds because the different electronegativities of C, O and N redistribute the partial charges to give an essentially zero dipole moment on the nitrile stretch (Socrates, 2005). However, moving the -OH group one position away from the -C≡N, partially restores the dipole on the nitrile stretch and its metallic odor character. A typical cyanohydrin without the nitrile odor character because of the predicted (Socrates, 2001) attenuation of its -C≡N vibration at 2150 cm^−1^ is 2-hydroxybutyronitrile. In contrast, 3-hydroxybutyronitrile, where the –OH group is moved away from the -C≡N, presents a metallic odor character to humans and yields a strong peak at 2150 cm^−1^ (Fig. 4*A*).

At the concentration used, flies were mildly attracted to 3-hydroxybutyronitrile (Fig. 4*B*) and mildly aversed by 2-hydroxybutyronitrile (Fig. 4*C*), both responses likely mediated by OR olfactory receptors because they are eliminated in *Orco^−^* mutants. To determine if flies respond to the nitrile odor character in the two hydroxynitriles, we trained them with footshocks to preferentially avoid either the aldehyde citronellal or citronellyl nitrile and tested them against 2- and 3-hydroxybutyronitrile. We expected that flies trained to avoid citronellyl nitrile will augment their avoidance of 3-hydroxybutyronitrile, but not of its isomer, with which they do not share a significant vibration at 2150 cm^−1^.

At the concentration used, naïve flies did not exhibit a preference between the hydroxynitriles (Fig. 4*D*, no shock). This indicates that differences in volatility or other properties cannot bias subsequent results. In confirmation, a similar lack of preference was observed for flies shocked in the presence of citronellal (aldehyde), which lacks the characteristic 2150 cm^−1^ vibration (Franco et al., 2011). Significantly however, flies shocked in citronellyl nitrile avoided 3-hydroxybutyronitrile (Fig. 4*D*). In the complementary experiment, naïve flies were mildly attracted to citronellyl nitrile as shown before (Fig. 1*B*; Franco et al., 2011) and maintained that mild preference if they were previously shocked in 2-hydroxybutyronitrile. In contrast, flies shocked in the presence of 3-hydroxybutyronitrile avoided citronellyl nitrile (Fig. 4*E*) in accord with the hypothesis that they would because the two odorants share the 2150 cm^−1^ vibration. Because the two odorants do not share any other features in common, our results suggest that flies identify their only common feature, the vibration at 2150 cm^−1^, which likely represents the nitrile odor character, to identify them as similar and selectively avoid them. Moreover, *Drosophila* do not selectively avoid 2-hydroxybutyronitrile, likely because they do not detect the -C≡N functional group when its vibration is attenuated. Collectively then the calculated IR intensity of -C≡N groups is predictive of whether *Drosophila* respond to the compound that bears nitrilic character, potentially paralleling human perception of these two odorants.

## Discussion

Experiments reported herein confirm independently the deuterium-nitrile cross-learning in *Drosophila* (Franco et al., 2011), with a different pair of isotopologues and a novel behavioral paradigm to test predictions of vibrational olfaction. Our results are consistent with the hypothesis that flies recognize and respond to odorant molecular vibrations in behaviorally salient contexts. Significantly, our current experiments establish two distinct advances. It was suggested (Block et al., 2015b; Vosshall, 2015) that results using deuterated odorants supporting vibrational olfaction in insects (Franco et al., 2011), were largely due to differences in isotopologue physical properties. Although experimental evidence does not support these critiques (Drimyli et al., 2016), our current behavioral experiments provide independent evidence consistent with a role for vibrational olfaction in *Drosophila* capitalizing on differences in the physical properties of normal, undeuterated compounds. Moreover, because the new behavioral assay requires recognition of common vibrational frequencies or other features in non-converging odorants, it is not sensitive to contaminants, volatility differences or other properties. Clearly, -CD/-CH can be easily discriminated even in the presence of a functional group such as -SH with a distinct intermediate vibrational frequency. This result indicates that flies resolve and respond independently to distinct vibrational modes of a single molecule and suggests in turn potential engagement of multiple olfactory receptors.

Is there a threshold for vibrational frequency differences beyond which divergence or convergence of physiologic and behavioral readouts reveal differences in odor salience? The resolution of the vibrational component of *Drosophila* olfaction in frequency and amplitude is hard to estimate. From purely physical considerations (Turin, 1996; Brookes et al., 2007), a thermal blurring width in detecting vibrational modes in the order of 200 cm^−1^ is expected. This may underlie at least in part, the -SH/-BH overlap, but as suggested by our results, this is not the case for -SH and either -C≡N or -CH. In the lower frequencies at the “fingerprint” region, resolution in amplitude rather than frequency is likely to be more important since odorants made of the elements C,H,O and N will all have modes in that region. Amplitude resolution is likely to depend to a large extent on signal processing in the brain, about which little is known at present. It is likely that processing in the brain sharpens resolution considerably, as it does for example in humans for color vision (broadly tuned cones but tens of thousands of colors discriminated) and hearing (broadly tuned cochlear activation, a few Hz perceptual resolution). Unfortunately, the stimulus flexibility in olfaction is not as continuous or easily accessible as in hearing and vision and it will be some time before we understand this. The 2- and 3-hydroxybutyronitrile experiments go some way in that direction and will serve as a benchmark for more accurate calculations of biological IETS line intensities. In fact, our collective results are likely to serve as useful constraints when developing models of biological IETS selection rules.

When an inelastic electron tunneling mechanism for vibrational olfaction was proposed twenty years ago, it clarified the features that would be required in order for a molecule to be detected and therefore have an odor (Turin, 1996). Insofar as tunneling electrons must interact with partial charges of the molecule, the model accounted for example for the fact that homonuclear diatomics (O_2_, N_2_) are odorless, as are molecules with very small partial charges such as CO. It appears that spectroscopic selection rules for the biological IETS suggested by our experiments resemble more closely those of IR spectroscopy than, say, those of Raman spectroscopy, where vibrational modes devoid of transition dipole are nevertheless detected by polarizability. Exact selection rules for IETS are the object of intense study (Solov'yov et al., 2012; Reese et al., 2016), but it appears reasonable to suppose that a functional group vibration undetectable in the IR would be at best weak in IETS. The well-known inability to detect the -C≡N stretch in cyanohydrins has been used to successfully design oxa-nitriles, -O-C-CN, as acid-stable ester replacements with only a barely perceptible nitrile character (Turin L, 2007, novel oxy-nitriles, Patent Application Flexitral, Inc. European Patent Application EP1687261 2007). In fact, the hydroxynitrile experiments provide mechanistic insights to odor character detection. The weak IR stretch of cyanohydrins does not elicit the characteristic nitrilic response, whereas moving the -OH “odotope” one carbon further, restores both IR intensity and the nitrilic character. This demonstrates a relation between odor and intensity of a single mode. In the case of 2-hydroxynitriles (cyanohydrins), the weakness of the IR peak, seen both experimentally (Socrates, 2001; Socrates, 2005) and computationally, does not appear to be due to a reduction in nitrile bond polarity per se, but to a change in the overall motion pattern of the atoms, which reduces dipole moment.

Our evidence indicates that *Drosophila* respond to boranes as if they were thiols possibly because their vibration differences are below resolution for the olfactory receptors engaged. Although they do not prove it, our data suggest that boranes may smell sulfuraceous to flies as has been reported for humans (Stock and Massenez, 1912; Anderson and Barker, 1950). It is possible then that the thermal blurring we suggest likely accounts for the equivalent responses of *Drosophila* to -SH and -BH may also underlie the not obvious reasons why humans perceive them both as sulfuraceous. However, the G protein-coupled serpentine human olfactory receptors are quite distinct from their channel-like dimeric counterparts in *Drosophila* and other insects (Vosshall, 2001; van der Goes van Naters and Carlson, 2007). If flies perceive boranes as sulfuraceous as humans do and given the evolutionary distance and differences in olfactory receptors, that would argue strongly that odor character is encoded in the odorant itself, not in its fit to receptors. Future experiments investigating the glomerular activation patterns in *Drosophila* antennal lobes on -SH and -BH exposure will provide a physiologic readout to address this hypothesis independently.

In the combinatorial shape theory (Malnic et al., 1999), if by some means the specificity of each receptor was changed without altering the projections of the olfactory neurons that bear them, the perceived odor would be completely different because the pattern of stimulation would be different. By contrast, vibration theory predicts that as long as the odorant binds to a sufficient number of receptors tuned to different parts of the vibrational spectrum, the perceived odor will be independent of the exact pattern of receptor activation. In other words, in the vibrational theory, odor character is encoded in the odorant itself, and the exact pattern of receptor binding is secondary. In this context, it is worth noting that the receptor data on *Drosophila* (Hallem and Carlson, 2006; Münch and Galizia, 2016) shows receptors responding to many odorants, and the response pattern is consistent with a reduction in dimensionality from fully combinatorial to a more compact, maximum-entropy code (Stevens, 2015). It is perhaps significant in this context that maximum-entropy coding has also been proposed as a mechanism for color constancy in human trichromatic vision (Clark and Skaff, 2009).

Therefore, we posit that collectively, our results provide evidence that odor character is encoded in the odorant itself, at least for those compounds tested. Together with previous *in vivo* studies on different insect species, these experiments strongly suggest that vibrational sensing is a critical component of insect olfaction.

## Synthesis

Reviewing Editor: Upinder Bhalla, National Center for Biological Sciences Decisions are customarily a result of the Reviewing Editor and the peer reviewers coming together and discussing their recommendations until a consensus is reached. When revisions are invited, a fact-based synthesis statement explaining their decision and outlining what is needed to prepare a revision will be listed below. The following reviewer(s) agreed to reveal their identity: Nixon Abraham

Synthesis:

This revision is much clearer as far as the data goes, and does establish

some behavioral findings for flies on a set of odorants. The presentation

continues to marred by strong assertions about the vibrational odor theory

being the basis for the observations, when these can at most be stated as being

consistent with the observations. Some of the affected lines are indicated.

The authors must restrict their conclusions to what the data supports,

and clearly separate observations from their inferences. The new

text adds such unhelpful overinterpretations in several places, and holds

back the paper.

Since these are relatively minor textual and presentation changes, this

is marked as a minor revision.

Specific points:

In the Significance Statement, Line 23: This sentence is incorrect. The paper

shows some behavioral results from a few odorants, It does not directly show

anything about vibrational frequencies, only that these may be consistent

with the behavior.

Line 193: Conclusion too sweeping. “Therefore, flies recognize the

2150 cm -1 vibration of the C-D or the C≡N 194 stretch and use it to guide

behavioral choices in two independent paradigms ((Franco et al. 2011) and

herein).”

It would be more correct to say that “this is consistent with...”

Similarly, line 196 should more carefully say something like “discriminated

efficiently between odors having C-D and C-H vibrations...”

- It would be nice to indicate significant comparisons where present in the

bar charts in all the figures. This is reported in the legend but the

conventional indication of significance in the charts would give additional

clarity.

- Please specify maze arm dimensions. This is needed to understand linear

flow rate from the given volume flow rate.

- Line 269 should be qualified because the naive flies clearly do differentiate

between the odorants, as seen from the no-shock cases in panel A and B.

Line 273: Presumably the avoidance cited here is for hexanol. It is a little

confusing because the numbers for benzhaldehyde are not given just above.

For consistency I suggest to move the statistics to the legend.

Line 283: Validates which response?

Legend 3C could be clarified a bit to explain what pairings are and are not

significant. Also see comment above regarding indicating significant comparisons

in the bar charts.

Line 287. 288: The data support the notion that the animals deem the odors

equivalent, but all one can say about the vibrations is that it is consistent

with the idea that this might be the basis for such a behavioral equivalence.

Line 306: Confusing phrasing of sentence “We selected...”

Line 329: ‘as expected... vibration’. This is a matter of interpretation and

should be removed or stated as being “consistent with...”

Line 330: ‘... based on ... ’ Again, this is a matter of interpretation and

the result itself simply should state what is observed, and at most should

suggest that it may be consistent with the common vibration that is postulated

to represent the nitrile odor character.

Line 348: “We show that...” This is only an interpretation, and must be

stated as such. What is shown are behavioral readouts of flies to a small

set of odors.

Line 353-354: “...our current experiments confirm independently...” This is

incorrect. The current experiments only present some behavioral observations

that may be consistent with a vibrational interpretation.

The block of lines 363-371 and 374-382 are duplicated.

line 450-453: The last sentence again overstates the implications of these

behavioral results and should be clarified to state that this is the

authors’ interpretation of the results.

Minor typos.

- Line 121: ‘was’ should be ‘were’

- Line 215: footschoks”. Also the phrasing of the sentence should be fixed.
